# Partial Anomalous Pulmonary Venous Return in Adults

**DOI:** 10.7759/cureus.26777

**Published:** 2022-07-12

**Authors:** Nourhan Chaaban, Hamna Shah, Akash Joshi, Shilpa Kshatriya

**Affiliations:** 1 Internal Medicine, University of Kansas School of Medicine, Wichita, USA; 2 Medicine, University of Kansas School of Medicine, Wichita, USA; 3 Cardiology, Heartland Cardiology, Wichita, USA; 4 Cardiology, University of Kansas School of Medicine, Wichita, USA

**Keywords:** atrial septal defect (asd), echocardiogram, pulmonary hypertension, superior vena cava (svc), partial anomalous pulmonary venous return (papvr), idiopathic pulmonary fibrosis (ipf)

## Abstract

Partial anomalous pulmonary venous return (PAPVR) is a spectrum of congenital cardiovascular abnormalities. It is most commonly found as an incidental finding. However, it can lead to severe pulmonary hypertension depending on the magnitude of the shunt involved. We report a case of a 60-year-old female patient with PAPVR detected incidentally on imaging. We aim to highlight the incidence of PAPVR in adults and to elaborate on its unique association with a duplicated superior vena cava.

## Introduction

Partial anomalous pulmonary venous return (PAPVR) is a spectrum of congenital cardiovascular abnormalities. The overall incidence of PAPVR is estimated to be 0.7 percent of the population [1]. In 1739, Winslow described this syndrome as a remaining embryonic connection between the systemic and pulmonary venous plexus [2]. The most common variant of this syndrome involves a connection between the right pulmonary vein and the systemic circulation involving either the superior vena cava (SVC) or the right atrium (RA); but it can also involve the coronary sinus, the inferior vena cava or the brachiocephalic vein [3]. PAPVR is uniquely found incidentally with no clinical symptoms.

## Case presentation

A 60-year-old female patient with prior comorbidities, including hypercholesteremia, hypothyroidism, and depression, presented for an annual physical examination. At her initial visit, the patient reported a chronic dry cough of four months duration, worse with exertion and relieved upon rest. The patient denied shortness of breath, chest pain, palpitations, orthopnea, or paroxysmal dyspnea. The patient was up to date on vaccinations. As for her social history, the patient reported no smoking or drug use.

She was concerned about her family history of lung disease and pulmonary hypertension. Her maternal uncle was diagnosed with idiopathic pulmonary fibrosis (IPF) at the age of 50. Her cousin on the mother's side had undergone a lung transplant recently after suffering from IPF since the age of 40. 

Physical examination, including vitals, was normal with flat jugular venous distension (JVD), symmetrical clear lung auscultation, and normal S1S2, regular rhythm with no murmurs on cardiac auscultation. An EKG showed normal sinus rhythm with right ventricular hypertrophy.

**Figure 1:**
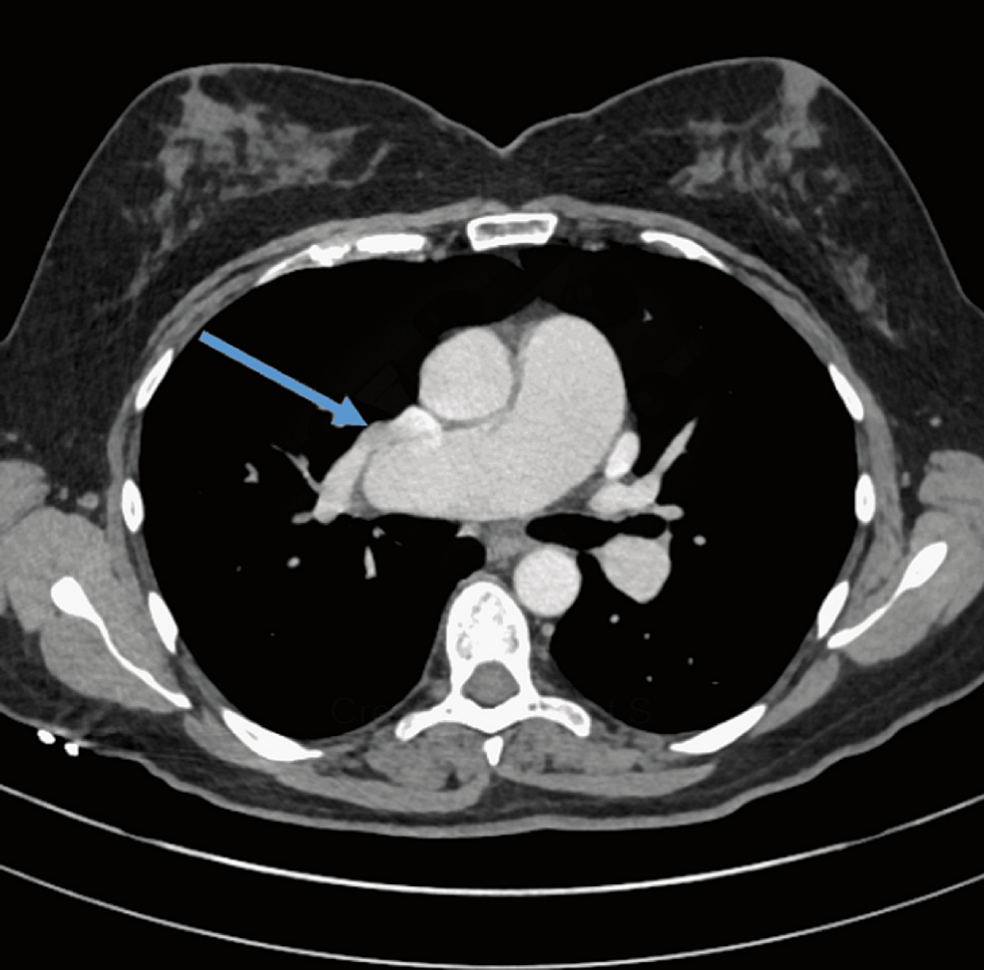
Chest CT scan with contrast showing PAPVR with the right upper and middle lobes draining into the right-sided SVC Arrow: right upper lobe vein draining in SVC PAPVR - partial anomalous pulmonary venous return; SVC - superior vena cava

Her chest CT scan with contrast showed PAPVR with the right upper and middle lobes connected into the right-sided superior vena cava (SVC) (Figure 1). This constitutes a left-to-right shunt. Also, a left-sided superior vena cava draining to the coronary sinus was noted. No pulmonary mass or consolidation was noted.

**Figure 2:**
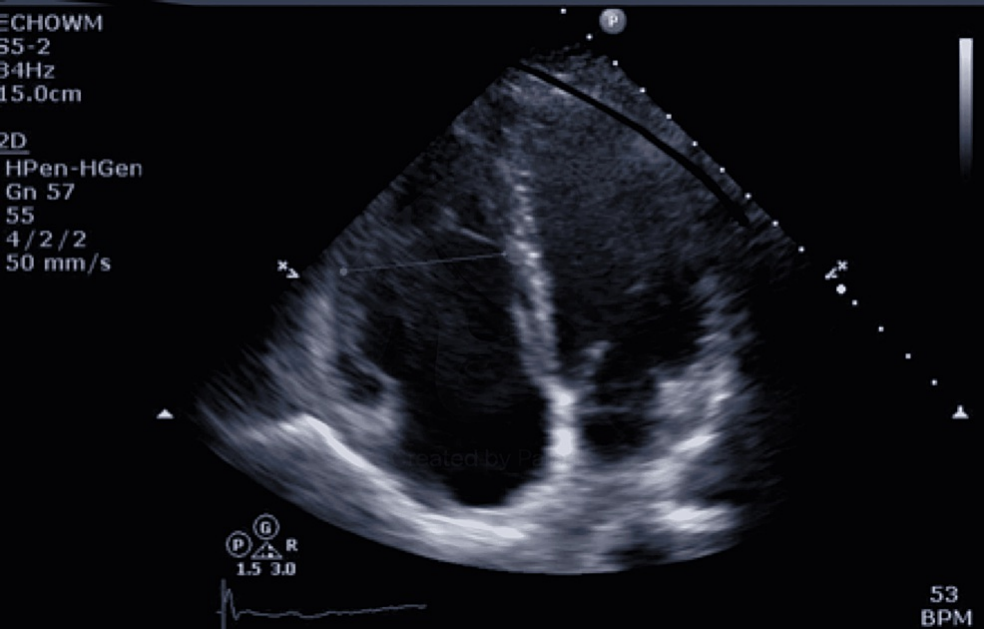
Echocardiogram showing mild dilatation of the right ventricle with a right ventricular systolic pressure of 28 mmHg

Her echocardiogram showed a normal left ventricular systolic function, an ejection fraction of 55-60%, and mild dilatation of the right ventricle with a right ventricular systolic pressure of 28 mmHg (Figure 2). Doppler findings showed no signs of pulmonary hypertension. Injection of contrast documented no obvious intracardiac shunt. A dilated coronary sinus with left superior vena cava was seen.

Subsequently, the patient was referred by the cardiology team to the cardiothoracic team for surgical management, where she underwent a modified Warden procedure. There was a creation of secundum atrial septal defect, enlargement of tiny sinus venous defect, patch closure of right superior vena cava-right atrium junction with patch pericardium, tricuspid valve repair, and creation of baffle between right upper and lower pulmonary veins to the left atrium. Her postoperative course was uneventful. She was discharged on 1L oxygen by nasal cannula for persistent nocturnal hypoxemia. She was treated with anticoagulation for three months and now takes aspirin. The patient went to cardiac rehabilitation after surgery for six weeks. She is currently doing well, with no restrictions. She is doing water aerobics which is going well for her. 

## Discussion

Partial anomalous pulmonary venous return (PAPVR) is a rare congenital abnormality that may be found incidentally in asymptomatic adults. It may have a wide spectrum of clinical presentations based on the shunt fraction. Also, when a significant fraction of the left-to-right shunt is left undiagnosed and unrepaired, the patient may develop pulmonary arterial hypertension (PAH). However, if there's an involving atrial septal defect (ASD), a shunt reversal could occur, resulting in Eisenmenger's syndrome with severe PAH [2].

Patients with PAPVR have a wide variety of clinical manifestations, including fatigue and dyspnea progressing to heart failure as well as recurrent pneumonia. Symptoms are usually dependent on the size of the left to right shunt and if there is an associated cardiac defect, including an atrial septal defect (ASD), for instance. In fact, the physiology of ASD is the same as that of PAPVR [4]. Moreover, the physical exam is clinically benign. However, cyanosis findings could be detected if there is a severe progression of the shunt. Also, a murmur of ASD could be found in case of its presence. Our patient was clinically asymptomatic, and PAPVR was identified incidentally with thoracic imaging.

In terms of the evaluation of PAH, transthoracic echocardiography (TTE) remains the gold standard diagnostic tool for identifying anomalous veins, while cardiac MRI and CT angiography (CTA) are superior in the evaluation of extracardiac vascular anatomy [5]. Moreover, cardiac MRI is a non-invasive diagnostic modality that enables the identification of the number of systemic veins, their origin, course, and site of drainage. In addition, cardiac MRI can detect atrial septal defects by identifying their type [6]. In our case, a TTE was performed, and it was sufficient for the diagnosis. 

The modality of treatment varies depending on the patients' presentation and symptoms. Surgical repair is recommended when there are symptoms caused by the shunt, when there is more than one anomalous vein involved, and if the shunt is moderate or large in severity. Hence, surgery would result in reducing the right ventricle (RV) size and pulmonary hypertension (PA) pressure. However, surgical repair can be challenging with the risk of causing thrombosis to the surgically operated vein [5]. The Warden procedure, reported in 1984, has been widely used for surgically correcting PAPVC, which connects to the SVC. Although the procedure has been effective in decreasing the risk of SVC or pulmonary venous obstruction, there have been a few complications after the procedure, including pulmonary venous obstruction, SVC obstruction, and sinus node dysfunction, warranting close serial follow-up [7]. In our case, a surgical approach was recommended by the cardiothoracic team due to the presence of symptoms related to the shunt fraction.

## Conclusions

In conclusion, PAPVR is a rare and often silent congenital anomaly that can have unusual presentation in adults. Prompt recognition and treatment are imperative to prevent the advancement of symptoms and development of severe PAH. CT chest, instead of a plain radiograph in patients with chronic cough, could be a better imaging modality to access for rare causes. Early surgical repair is the treatment of choice in symptomatic patients. On the other hand, in cases with severe pulmonary hypertension, earlier therapies, including an aggressive pulmonary vasodilator therapy, such as bosentan, could be considered. 
